# Choosing a nursing specialty: connection to nursing students’ personality traits, clinical self-efficacy, adoption of technology changes, and specialty prestige; a cross-sectional study

**DOI:** 10.1186/s12912-024-01813-3

**Published:** 2024-03-04

**Authors:** Lilach Ben Shabat, Michal Itzhaki

**Affiliations:** Nursing Department, School of Health Professions, Faculty of Medical and Health Sciences , Tel Aviv University , Tel Aviv, Israel

**Keywords:** Clinical self-efficacy, Nursing specialty, Nursing students, Personality traits

## Abstract

**Background:**

Choosing a field of specialization within the nursing profession is affected by nurses’ personality traits, self-confidence in performing clinical skills, and the field’s prestige. A successful choice of area of expertise may improve nurses’ job satisfaction and reduce job mobility. This study aims to examine the relationship between personality traits, clinical self-efficacy, perceived prestige, adoption of technological changes, and choice of specialty field among nursing students.

**Methods:**

A cross-sectional study was conducted. One-hundred-twenty-seven undergraduate nursing students in their fourth year of studies at a large university in Israel participated in the study. The questionnaire administered was comprised of six parts: demographic data, personality traits, adoption of technological changes, clinical self-efficacy, perceived prestige, and intention to select a field of specialization.

**Results:**

Acute disciplines were rated more prestigious than chronic disciplines, with intensive care and emergency medicine considered the most prestigious, while mental health and geriatrics were the least prestigious. Students’ mean perceived confidence in performing nursing clinical skills was high and more than half considered themselves open to technology changes. Positive correlations were found between prestige and intention to choose a field of expertise (*r* = 0.41, *p* < 0.001) and the personality trait of openness and the intention to choose an acute care area (*r* = 0.26, *p* < 0.01).

**Conclusions:**

Despite the gradual aging of the population and the increase in chronic morbidity, which demand a greater nursing focus on older adults, and notwithstanding the mental health reforms, nursing students perceive geriatrics and mental health as less prestigious fields. A career development path can be applied by developing a tool for occupational guidance designed to rank students’ suitability for specialty fields and thus help them choose the area that best suits them.

**Supplementary Information:**

The online version contains supplementary material available at 10.1186/s12912-024-01813-3.

## Introduction

With constant advances in medicine and the increasing complexity of patient’s medical conditions in the community and in the hospital, nursing as a profession has adapted to the new circumstances. Registered nurses (RNs) take one of the following education paths: a bachelor’s degree in nursing, an associate’s degree in nursing, or a diploma from an approved nursing program. Their expertise, responsibilities, and everyday routine are usually determined by the work environment and the population under their care [1]. Nursing is comprised of two prominent areas of expertise: chronic and acute care. The acute care field includes intensive care units, emergency medicine, operation rooms, and delivery rooms. The chronic care field relates to departments such as internal care, surgical, mental health, geriatric, and dialysis [2].

### Choosing an area of expertise within the nursing profession

Many possibilities exist for RNs’ work as specialists with specific patient groups, for example, in the fields of addiction, cardiovascular, critical care, public health, genetics, neonatology, nephrology, and rehabilitation. Advanced Practice RNs (APRNs) are clinical nurse specialists who have completed a Master’s degree that qualifies them to provide direct patient care in nursing specialties such as anesthesia and midwifery [1].

In Israel, nurses who pass the certification exam for RNs can choose to specialize in one of 26 areas of advanced training. Advanced training courses provide nurses with professional knowledge and specific skills in specific areas of expertise such as intensive care, emergency medicine, operating room, geriatrics, mental health, preterm infants, and pediatric intensive care [3].

Previous studies that examined nursing students’ choice of specialization field found that there are common time points in the training process when they formulate their decision regarding their desired field of specialty. Hunt et al. [4] examined the career interests of 564 nursing students at the University of Auckland in New Zealand over a ten-year period, upon entering the training program and upon completing the program. At the beginning of their nursing studies, the highest interest was in emergency care and child health, and the least interest was in older persons’ health. At the end of the training program, the highest interest was still in child health as well as in surgery, while older persons’ health remained of the least interest.

Toren and Zelker [5] examined the factors that affect the choice of workplace among 36 fourth-year nursing students at a major Israeli university. They found that the students considered four aspects when making their decision: choosing between working in a hospital or a community-based medical clinic, choosing a hospital department, choosing a specific hospital, and the perception of the ideal hospital to work in, which includes a combination of satisfactory working conditions, geographic proximity, and a pleasant social atmosphere.

### Personality traits

The five-factor model developed by McCrae and Costa McCrae [6] addresses five categories of traits, each consisting of various sub-traits: (1) Extraversion: characterized by a high social need, ability to connect, assertiveness, a tendency to talkativeness and activeness. (2) Neuroticism: characterized by anxiety, depression, anger, embarrassment, worry, emotions, and insecurity. (3) Agreeableness: characterized by kindness, flexibility, confidence and trust, a convenient temperament, a cooperative nature, a capacity to forgive, and tolerance. (4) Conscientiousness: characterized by caution, thoroughness, responsibility, organization and planning, hard work, perseverance, and self-discipline. (5) Openness to experience: characterized by an active imagination, curiosity, wide horizons, high intelligence, and artistic sensitivity.

Kennedy et al. [7] reviewed 13 articles published between the years 1965 and 2010 and found that similar personality traits can be identified among nurses who choose to work in a specific field. Studies profiling the personality of nurses working in the departments of emergency medicine, oncology, and nephrology found that the majority were task-oriented, independent, hardworking, preferred to work alone and maintained control over their work environment. Nurses working in the emergency medicine department were identified by extroversion, openness to change, pleasantness, high impulsivity, high competence, and low sensitivity. This review suggests that a good match between personality traits and the area of ​​specialization may improve nurses’ work efficiency, job satisfaction, and reduce attrition and job mobility. Regarding nursing students, Berl [2] examined 67 nursing students in their final year of studies and found differences between the personality traits of students who chose different areas of specialization: Conscientiousness stood out among the students who chose to work in pediatrics and in intensive care units, and agreeableness was a prominent trait among students who chose to work in the field of obstetrics and gynecology.

### Clinical self-efficacy

Bandura [8] defined self-efficacy as a person’s evaluation of the ability to organize and perform actions that are required to complete a task. In 2001, Bandura [8] added that self-efficacy is one’s perceived success or failure in performing challenging or complicated tasks. Oetker-Black, Kreye, Davis, Underwood and Naug [9] developed the concept of clinical self-efficacy, which refers to self-confidence in making clinical decisions and performing clinical nursing skills.

### Prestige of the specialty field

Studies examining the relationship between perceived prestige and the choice of field of specialization were mostly conducted among medical students. In a study conducted with medical students from five universities in Austria and Switzerland, based on status and socioeconomic aspects, they rated prestige as one of the main factors influencing their choice of field of specialization. Prestige was mainly related to the desired work environments of specialists in private practice [10]. A study conducted in Turkey among resident physicians at the Medical Specialization Examination found that the common reasons for choosing the specialty were career opportunities and working conditions [11].

### Adoption of technological advancements

The tendency to adopt innovations and technologies differs among people. Technological changes, innovations or inventions have been defined as an idea, a procedure, or a system that is perceived as novel by those who adopt them [12]. According to Rogers [12], there are five categories in which people are classified regarding the adoption of innovations: (1) Innovators: the first to adopt innovations. Those belonging to this group are bold individuals who are not deterred by risks. They constantly seek innovations and are quick to adopt them (2.5% of the population). (2) Early adopters: are the second to embrace innovations. They are open to change and are considered opinion leaders (13.5% of the population). (3) Early majority: Tend to adopt innovations at a changing rate, usually with the support of the early adopters (34% of the population). (4) Late majority: Tend to adopt innovations later than the rest of the population. They regard innovations with great suspicion (34% of the total population). (5) Laggard: The last to adopt novelties. They are reluctant to innovate and change, they prefer to adhere to the old and familiar (16% of the population). Despite the differences in the rate of adoption, the innovations will eventually be adopted [12].

Bunpin, Chapman, Blegan and Spetz [13] examined the implementation of innovative behavior among RNs in nine California hospitals and found that those who tended to adopt novelties are nurses who work in management positions and nurses with a high level of education, since they often need problem-solving skills.

Stilgenbauer and Fitzpatrick [14], who examined the innovativeness of nurse leaders in acute care settings, concluded that understanding the degrees of innovativeness may help nurse leaders lead and innovate changes to enhance patient outcomes.

The aim of the current study was to examine the relationship between personality traits, clinical self-efficacy, perceived prestige, adoption of technological changes, and the choice of field of specialization among nursing students.

## Methods

### Design

A quantitative descriptive, cross-sectional study was conducted.

### Data collection

The study was conducted among undergraduate nursing students in their final year of studies (fourth year) at a large university located in central Israel. The questionnaires were printed and distributed manually. Of 209 students enrolled in the fourth year of the nursing program, 127 completed a questionnaire and signed an informed consent form (response rate 61%). The questionnaires included in the study were those that were completed in full. To reach a larger number of participating students, an additional date was scheduled to distribute questionnaires, but it was canceled due to the outbreak of the COVID-19 pandemic. Data collection lasted three months. The sample size was calculated to match a power of 80%, an effect size of 0.4, and a significance level of 0.95. The calculation showed that the size required was at least 100 respondents. This calculation was performed with the G*Power 3.1 software [15].

### Instruments

The questionnaire administered was comprised of six parts:

#### Demographic data

Demographic data were collected, including age, gender, religion, marital status, education and the field in which they wish to work as RNs.

### Personality traits

Personality traits were measured with the Big Five Index (BFI) [16], where respondents were asked to rank their agreement with 44 items representing different traits. Each item begins with the phrase “I see myself as…”. Items are ranked on a 5-point Likert scale, from 1 “strongly disagree” to 5 “strongly agree”. A separate score is calculated for each trait by averaging the items that pertain to it. The items are divided into 5 personality traits: Extraversion (8 items), Neuroticism (8 items), Agreeableness (9 items), Conscientiousness (9 items), and Openness to Experience (10 items). All five traits were shown to have convergent and discriminant validity across an adjective scale and questionnaire measures and to endure across decades in adults [6]. The Cronbach’s alpha score of the BFI is reported as 0.83 [16].

The questionnaire was translated into Hebrew by Etzion and Lasky [17]. The reliability of the items in the Hebrew version is: Extraversion α = 0.80; Neuroticism α = 0.81; Agreeableness α = 0.68; Openness to Experience α = 0.76; and Conscientiousness α = 0.73. The reliability of the items in the current study was: Agreeableness α = 0.67; Extraversion α = 0.61; Neuroticism α = 0.72; Openness to Experience α = 0.73; and Conscientiousness α = 0.62.

### Adoption of technological changes

The questionnaire was developed by Hurt, Joseph and Cook [18] and consisted of 20 statements. The respondents were asked to rate their level of agreement with each statement on a Likert scale ranging from 1 “agree” to 5 “strongly disagree”. Each participant’s innovation score was calculated in three steps. Step One: The participant’s ranking of items 4,6,7,10,13,15,17,20 was summed. Step Two: Responses for items 1,2,3,5,8,9,11,12,14,16,18,19 were summed Step Three: Forty-two points were added to the sum calculated in the second step and the sum received in the first step was subtracted. A score above 80 classifies the respondent as an Innovator, 69–80 are Early Adopters, 57–68 are Early Majority, 46–56 are Late Majority, and a score lower than 46 classifies respondents as Laggards. The questionnaire was translated into Hebrew by a full professor from the Department of Nursing at Tel Aviv University using the back-translation method [19]. High and acceptable reliability and discriminant validity levels were reported [18]. The questionnaires’ reliability was reported as α = 0.82 [18], and in the present study it was α = 0.76.

### Clinical self efficacy

Assessment of clinical self-efficacy was conducted with the Clinical Self-Efficacy Scale (CSES) [9]. This scale consists of the following nine nursing clinical skills: performing intramuscular and subcutaneous injections, replacing sterile dressing, inserting Foley catheter, inserting a nasogastric tube, starting an intravenous line, enabling patients to move from a bed to a chair, preparing intravenous piggyback, and administrating tube feeding by Percutaneous Endoscopic Gastrostomy (PEG).

In the present study, we deleted the skill of PEG since students in Israel do not perform it. The other clinical skills presented in the questionnaire are common in Israeli health systems as part of chronic and acute patient care. Permission to use this questionnaire was granted to us by Sharon L. Oetker-Black on December 11th, 2018. The questionnaire was translated into Hebrew using the back-translation method [19]. Clinical self-efficacy was tested by the participants’ perceived confidence in performing each of the clinical skills, for example: “How confident are you right now that you will be able to perform an intramuscular injection?“. This was measured on a scale of 1–10, where 1 meant “no confidence” and 10 meant “total confidence”. In addition, we added a question about the extent of experience with each of the skills, for example: “To what extent have you performed subcutaneous injections?” ranked on a scale of 0 (not at all) to 6 (often). A higher mean score indicated higher clinical self-efficacy.

A Content Validity Index (CVI) was used to rate the relevance of each item using a four-point scale. All items on the CSES received a rating of 3 or 4 from experts (undergraduate nursing faculty who actively taught the clinical skills contained in the CSES) who rated the items as quite/very relevant [9]. Construct validity was established by comparing groups on three designated clinical skills and comparing their scores using t-tests to assess whether significant differences in the CSES scores were noted between students who performed or did not perform the clinical skill [9].

According to Oetker-Black et al. [9], the instrument’s reliability is α = 0.90, and in the present study it was α = 0.85.

### Perceived prestige

An index was developed by the current researchers to examine the perceived prestige of nine departments that represent different areas of specialization in nursing: intensive care units, emergency medicine, operating room, internal care and surgery departments, delivery room, mental health, geriatrics, dialysis units, and community health (primary medicine). Prestige was rated on a scale of 1 “not prestigious at all” to 10 “very prestigious”. The reliability was α = 0.60.

### Intention to select a field of specialization

Students’ intention to choose different areas of ​​specialization of the nine areas mentioned above was ranked on a scale of 1 “not interested at all” and 6 “very interested”.

### Data analysis

Data were analyzed using SPSS statistics software for windows (version 27; IBM, Armonk, NY). Descriptive statistics were used for the demographic and research variables. Correlations between research variables were calculated using Pearson coefficient tests. To predict whether clinical self-efficacy mediates the relationship between perceived prestige and preference for field of specialization, a simple two-step regression analysis was performed. Statistical significance was taken at *p* < 0.05. The STROBE checklist was used (Supplementary File 1).

## Results

### Participant characteristics

Participants were 127 nursing students in their final year of studies (fourth year), studying at six nursing schools affiliated with the Department of Nursing at a large university in central Israel. The demographic characteristics of the participants are presented in Table 1. The mean age was 24.26 years (SD = 3.002). The majority were women (77%) and single (80.2%). About 60% were Jewish and about 40% were Israeli Arabs (Muslim, Christian, Druze). Only 7.1% of the participants were ultra-orthodox religious, 46.8% were traditional, and 46% were secular.

**Table 1:** Distributions of participants’ demographic variables (*N* = 127)

Variable	Category	N	%
**Gender**	Male	29	23
	Female	97	77
**Marital status**	Single	101	80.2
	Married	20	15.9
	In a relationship	5	4
**Birthplace**	Israel	109	86.5
	Former Soviet Union	10	7.9
	Other	7	5.6
**Ethnicity**	Jewish	75	59.5
	Arab Muslim	42	33.3
	Arab Christian	5	4
	Druse	2	1.6
	Other	2	1.6
**Religiosity**	Ultra-orthodox	9	7.1
	Traditional	59	46.8
	Secular	58	46

### Research variables: descriptive statistics

Regarding perceived prestige, a higher mean of prestige was measured for acute care (M = 8.27, SD = 1.27) than for the chronic care specialization areas (M = 4.55, SD = 1.65), on a scale ranging from 1 to 10 (t(124) = 20.93, *p* < 0.001, d = 2.53). Regarding students’ intention to choose to work in an acute care discipline versus in chronic care, the mean of intention to choose acute care was higher (M = 3.78, SD = 1.14) than that of chronic care (M = 2.52, SD = 0.87) (t(124) = 9.95, *p* < 0.001, d = 1.25).

**Fig. 1:**
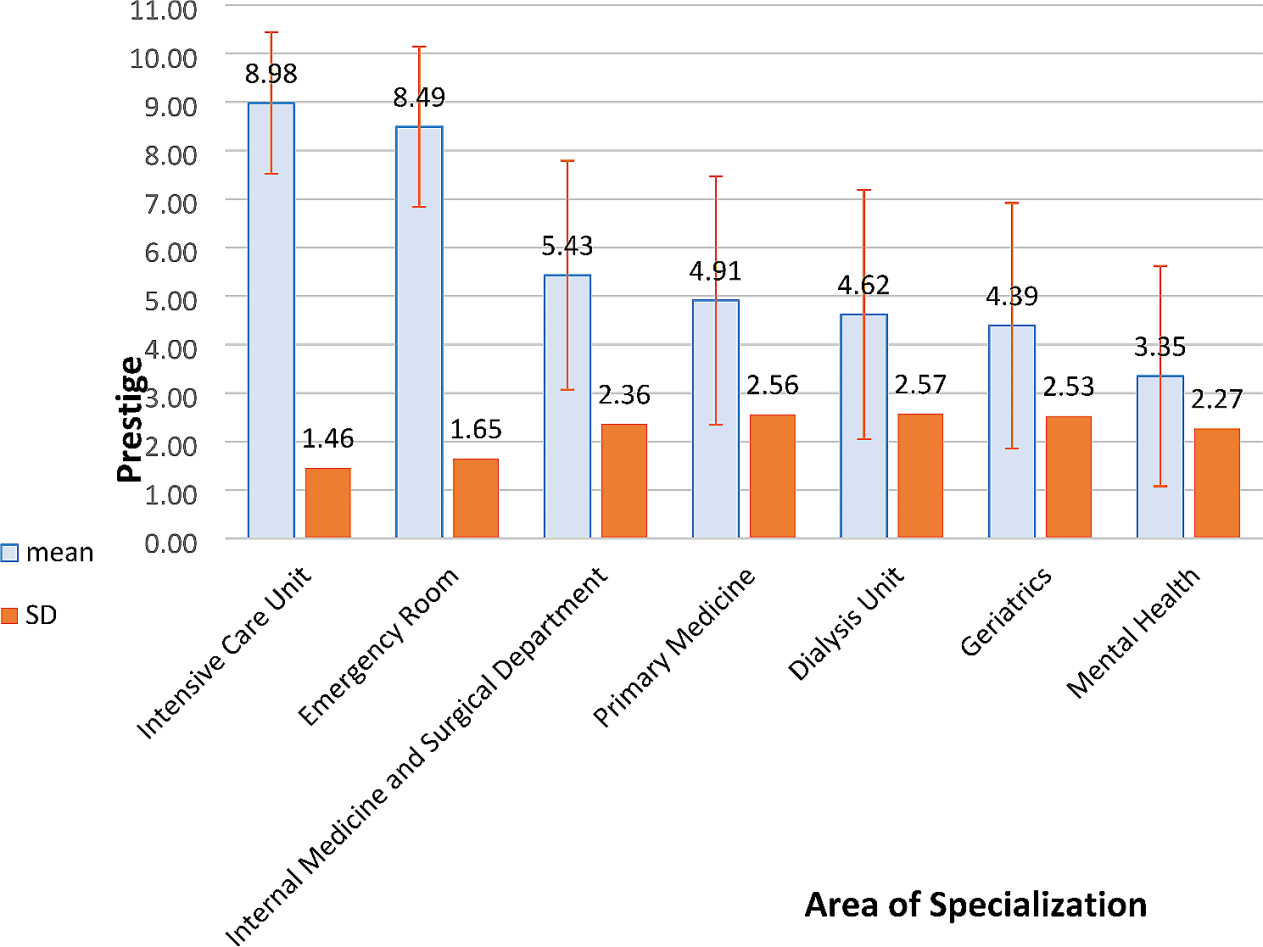
Description of the different areas of specialization by their prestige

Among the acute care specialization areas, the most prestigious were intensive care (M = 8.98, SD = 1.46) and emergency medicine (M = 8.49, SD = 1.65) (F(3, 372) = 12.16, *p* < 0.001, η^2^ = 0.089). Among the chronic care specialization areas, the most prestigious were the internal medicine and surgical departments (M = 5.40, SD = 2.35), whereas geriatrics (M = 4.39, SD = 2.53) and mental health (M = 3.35, SD = 2.27) were at the bottom of the list (F(4, 492) = 17.12, *p* < 0.001, η^2^ = 0.122) (Fig. 1).

The mean score for the adoption of technological innovations was 3.26 and the standard deviation was 0.38 (the range was 2.3 to 4.2). Also, 60% of the students belonged to the “Early Majority” category, 28% to the “Early Adopters”, 9.5% to the “Late Majority”, and 2.5% to the “Innovators”. No students were classified as “Laggards”. Regarding clinical self-efficacy, the mean perceived confidence in performing each of the clinical skills was higher (M = 6.83, SD = 1.88, with a range of 1 to 10) than the experience with these skills (M = 3.74, SD = 1.05, with a range of 1 to 6).

### Associations between the research variables

Positive correlations were found between the following pairs: between the perceived prestige of a field of specialization and the intention to choose it (*r* = 0.41, *p* < 0.001) and between the prestige of a field of specialization and the personality trait of openness (*r* = 0.76, *p* ≤ 0.05). Weak positive corrections were found between clinical self-efficacy ( the extent of experience with nursing clinical skills) and intention to choose a field of specialization (*r* = 0.18, *p* ≤ 0.05), clinical self-efficacy (confidence in performing clinical skills), and personality trait of extroversion (*r* = 0.21, *p* < 0.05), the extent of experience with nursing clinical skills, and the personality traits of extroversion (*r* = 0.20, *p* < 0.05), agreeableness (*r* = 0.19, *p* < 0.05), and conscientiousness (*r* = 0.22, *p* < 0.05). A positive strong correlation was found between the extent of experience with nursing clinical skills and the personality trait of openness (*r* = 0.91, *p* < 0.05) (Table 2).

**Table 2:** Pearson correlation matrix between variables (*N* = 127)

Variables		1	2	3	4	5	6	7	8	9
BIG 5	1. Openness	--	--	--	--	--	--	--	--	--
	2. Extraversion	**0.42** ^***^	--	--	--	--	--	--	--	--
	3. Agreeableness	0.07	0.19^*^	--	--	--	--	--	--	--
	4. Conscientiousness	0.19^*^	**0.52** ^***^	**0.45** ^***^	--	--	--	--	--	--
	5. Neuroticism	0.10	− 0.10	**− 0.29** ^**^	− 0.16	--	--	--	--	--
6. Adoption of innovations		**0.42** ^***^	**0.42** ^***^	0.08	0.16	0.14	--	--	--	--
Clinical self-efficacy	7. Extent of experience with clinical skills	**0.91** ^*^	**0.20** ^*^	**0.19** ^*^	**0.22** ^*^	− 0.01	0.11	--	--	--
	8. Confidence in performing clinical skills	0.15	**0.21** ^*^	0.221	0.2	-0.1	0.15	**0.73** ^**^	--	--
9. Prestige		**0.76** ^*^	0.03	0.11	0.16	0.05	0.12	0.08	0.10	--
10. Intention to choose specialty area		0.17	0.05	0.10	0.09	0.06	0.07	**0.18** ^*^	0.15	**0.41** ^***^

*p* < 0.05 ^*^, *p* < 0.01 ^**^, *p* < 0.001 ^***^

No significant correlation was found between the five personality traits and the intention to choose specific fields of specialization. A multivariate analysis of variance (MANOVA) conducted revealed that the personality trait with the strongest effect on the choice between acute care and chronic care disciplines was openness. Regarding the other four personality traits, no between-subject effects were significant [F (1,74) ≤ 1.4, *p* ≥ 0.2, ɳ2 ≤ 0.019]. Pearson’s correlation analyses yielded a significant moderate positive correlation between the openness personality trait and the intention to choose an acute care area of ​​specialization (*r* = 0.26, *p* < 0.01).

Pearson’s correlation analyses between the perceived prestige of areas of specialization by the nine types of departments, and the intention to choose acute care versus a chronic care discipline and clinical self-efficacy, showed significant positive moderate correlations between intention to choose acute care and perceived prestige, among four acute care specializations: intensive care (*r* = 0.29, *p* < 0.001), emergency medicine (*r* = 0.27, *p* < 0.01), operating room (*r* = 0.26, *p* < 0.01), and delivery room (*r* = 0.29, *p* < 0.01). However, between the intention to choose a chronic care field and the perceived prestige of the specialization area, significant positive relationships were found with the following domains: dialysis unit (*r* = 0.24, *p* < 0.01), mental health (*r* = 0.28, *p* < 0.01), community health (*r* = 0.41, *p* < 0.01), and geriatrics (*r* = 0.38, *p* < 0.001), meaning that the more prestigious the perception of the field, the greater the tendency to choose to specialize in it.

A weak significant negative correlation was found between the intention to choose to work in the chronic field and the perceived prestige of the acute care area of specialization in the emergency medicine unit (r = -. 21, *p* < 0.05). That is, students who rated emergency medicine as more prestigious had a weaker inclination towards the chronic fields. Between clinical self-efficacy and the perceived prestige of specific areas of specialization, two significant correlations were identified: a weak positive correlation with emergency medicine (*r* = 0.18, *p* < 0.05) and a moderate negative correlation with the internal care and surgery departments (*r*=-0.26, *p* < 0.01). This means that students with high clinical self-efficacy rated emergency medicine as prestigious, while students with low clinical self-efficacy rated the internal care and surgery departments as prestigious (Table 3).

**Table 3:** Pearson correlations between perceived prestige of specialization area, clinical self-efficacy, and intention to choose acute/chronic discipline

Perceived prestige of area of specialization		Clinical self-efficacy	Intention to choose chronic discipline	Intention to choose acute discipline
Acute Discipline	Intensive Care Unit	0.06	− 0.16	**0.29** ^***^
	Emergency Room	**0.18** ^*^	**− 0.21** ^*^	**0.27** ^**^
	Operating Room	0.07	− 0.02	**0.26** ^**^
	Delivery Room	0.08	0.17	**0.29** ^***^
Chronic Discipline	Internal Care and Surgery Departments	**− 0.26** ^**^	− 0.06	0.00
	Dialysis Unit	0.03	**0.24** ^**^	0.16
	Mental health	0.12	**0.28** ^**^	0.12
	Community health	0.17	**0.41** ^**^	− 0.07
	Geriatric	0.03	**0.38** ^**^	0.09

*p* < 0.05 ^*^, *p* < 0.01 ^**^, *p* < 0.001 ^***^

Regarding ethnicity, a significant relationship was found between intention to choose the acute care discipline and ethnicity, with Israeli Arab students being more likely to prefer acute care departments than Jewish students. A multivariate analysis of variance was performed, with ethnicity as the between-subject variable and inclination towards acute versus chronic care as the within-subject variable; The multivariate analysis of variance yielded a significant main effect [F_(2,122)_ = 3.52, *p* = 0.033, ɳ^2^ = 0.054]. The between-subject effect was significant only for the acute care preference [F _(1,123)_ = 6.26, *p* = 0.014, ɳ^2^ = 0.048], with the mean score for intention to choose acute care specializations being higher among Israeli Arab students.

Regarding marital status, a significant relationship was found between intention towards acute care and marital status, with single students being more likely to prefer acute care departments than those in a relationship/married. A multivariate analysis of variance was performed, with marital status as the between-subject variable and intention as the within-subject variable; The multivariate analysis of variance yielded a marginally significant main effect [F_(2,122)_ = 2.62, *p* = 0.077, ɳ^2^= 0.04]. The between-subject effect was found to be significant only for acute care preference [F_(1,123)_ = 4.86, *p* = 0.029, ɳ^2^= 0.038], and the mean score of inclination towards acute care specializations was found to be higher among singles. In addition, we examined whether clinical self-efficacy mediates the relationship between perceived prestige and preference for the field of specialization. Two simple linear regressions were performed. In the first regression, perceived prestige was the independent variable, and intention to choose a specialty area was the dependent variable. The regression revealed that perceived prestige was statistically significantly associated with the intention to choose a specialty area (β = 0.41, *p* ≤ 0.001). The regression explained 16.8% of the variance (Table 4, model A). In the second regression, perceived prestige was the independent variable and clinical self-efficacy the dependent variable. The regression revealed no statistically significant relationship between the variables, meaning that clinical self-efficacy does not mediate the relationship between perceived prestige and specialization preference (Table 4, model B).

**Table 4:** Two simple linear regressions (model A and model B) to examine the mediation effect

	Independent Variable	Dependent Variable	B	SE	β	R^2^ (%)
Model A	Perceived prestige	Intention to choose specialty area	0.26	0.05	0.41***	16.8
Model B	Perceived prestige	Clinical self-efficacy (extent of experience with clinical skills)	0.17	0.15	0.11	10.0

***p < 0.001

## Discussion

The present study examined the relationship between personality traits, perceived prestige of fields of specialization, adoption of technological change, and clinical self-efficacy, and the intention to choose a field of specialization among nursing students. The research findings showed that among nursing students, the perceived prestige of the acute care specializations was twice higher than that of the chronic fields. Among the acute fields, the most prestigious was intensive care, whereas among the chronic fields the most prestigious were the internal medicine and surgery departments, while mental health and geriatrics were the least prestigious. Similarly, Hindhede and Larsen [20] examined the prestige ratings of areas of specialization among physicians, nurses, medical and nursing students. They found that the different sectors have similar perceived prestige. The most prestigious areas were those that involved saving lives and those requiring highly complex treatments, such as surgery and anesthesiology. Pediatrics was considered prestigious due to the importance attributed to saving children’s lives. However, mental health and geriatrics were considered to have low prestige. Mental health was identified with long-term treatment, difficulty measuring the degree of recovery and stigmatic complicated patients, and geriatrics was considered a less “sexy” field. These findings are alarming considering the international shortage in healthcare professionals in geriatrics and mental health.

In mental health, according to the Health Resources and Services Administration [21], in 2018 the total pool of workers in the US was estimated at only 66,740 professionals and a shortage of 5,124 mental health professionals was reported. By the year 2030, the demand for mental health professionals is expected to exceed the supply by 16,450 personnel. The ratio per capita of mental health nurses in the US in 2018 was 6.98 per 100,000 people [22].

In Israel, a mental health reform implemented in July 2015 emphasized the need to increase the quantity of healthcare provided within the community and, as a result, the need to train additional personnel to work in community clinics [23]. Nevertheless, in 2018 there were only 1,403 nurses in Israel with basic mental health training, and the proportion of nurses working in mental health was only 0.12 per 1,000 people [24].

In the field of geriatrics, the number of people aged 65 and older in the world reached 616 million in 2015 and they constituted 9% of the total population. By 2030, the number of people aged 65 and over is expected to reach 999 million, which will constitute 12% of the world’s total population [25]. Despite the increasing life expectancy and the aging of the population, in the US only 2.6% of advanced practice registered nurses choose to specialize in geriatrics, and less than 1% of RNs choose geriatrics as their specialty [26]. Lack of motivation, knowledge, and willingness to choose this field as an area of ​​expertise are evident among nurses [26, 27].

In Israel, at the end of 2018 the population was estimated at about 8.9 million, and the population of people aged 65 and over at about one million. The latter had increased not only in absolute numbers but also in terms of their part in the total population, which more than doubled since 1955, when it was 4.8% of the total population compared to 11.8% at the end of 2018 [28]. Despite this, in 2018 there were only 1,548 nurses with basic training in the field of geriatrics in Israel, while the proportion of nurses working in geriatrics was 0.13 per 1,000 people [24].

In the current study, students with high clinical self-efficacy chose to specialize in acute care fields considered more prestigious. Students with high self-efficacy rated emergency medicine, classified as acute, as prestigious, while students with low self-efficacy rated the field of internal medicine and surgery departments, a chronic care field, as prestigious. Perhaps when students perceive themselves as capable, they have more confidence to choose ​​emergency medicine, a field that is sometimes considered complex [29].

The assessment of students’ clinical self-efficacy was examined in the present study through self-confidence in the ability to perform nursing clinical skills and the extent of experience with procedures. The study showed that despite having limited experience with procedures, the students expressed confidence in performing them. This may be due to practicing the clinical skills during simulations, which occupy 20% of clinical training, in accordance with the core curriculum of the Nursing Division at the Israeli Ministry of Health [30]. Simulations conducted under the supervision of a clinical instructor promote students’ self-confidence in performing nursing skills [31] and improve self-efficacy, perceived competence, and satisfaction with studies [32]. Thus, the use of simulations in less commonly performed skills may improve their perception of clinical self-efficacy.

Regarding ethnicity, it was found that Israeli Arab students tended to choose acute care departments and to rate the chronic field as more prestigious than did Jewish students. In a study by Popper-Giveon, Liberman and Keshet [33], the main motive for choosing the medical profession among Arab physicians in Israel was practical rather than altruistic, as medicine is perceived as a source of substantial and secure income in the Israeli labor market and as a domain in which they will not be discriminated against due to their ethnic identity. Another motive was the integrated Jewish-Arab work environment. Hospitals and medical clinics are arenas with less disparities between Arabs and Jews. Physicians viewed practicing medicine as a way of helping reduce the health gaps between the Jewish majority and the Arab minority. These findings might explain the choice of Arab nursing students to specialize in the internal-surgical field. Choosing this field allows them to gain experience, learn about a wide range of chronic diseases, and obtain broad knowledge, all of which can be applied in the treatment of the Arab population and thus reduce health disparities.

In the present study, the openness personality trait was found to be related to the choice of the acute care discipline. Working as a nurse in acute care departments poses unique professional challenges and demands, such as caring for complex patients, using advanced technology, and making decisions in a fast pace and a stressful atmosphere [2]. The personality trait of openness has been found to help nurses in the acute field care for patients from diverse backgrounds in an unexpected and hectic work environment [34].

As for the variable of adoption of technological change, the present study found that most research participants (60%) belonged to the “Early Majority” category, meaning that they tended to adopt innovations at a variable rate, and no participants fit the “Laggard” category. This finding can be explained by the fact that the students belong to Generation Z, which makes up 27% of the global population. They were born into the digital world and technology is an inherent part of their life [35].

Notably, the current study was conducted during the period of COVID-19, when the intensive care units and the general hospitals received considerable media coverage, with great appreciation expressed towards the healthcare teams. Health professionals, especially nurses, were seen as the heroes of modern times [36]. Therefore, the nursing students’ perceived high prestige of the acute care specializations may have been affected by a bias during the Covid-19 period. Further studies should examine nursing students’ choice of a nursing specialty in times of routine.

### Limitations

The self-administered questionnaire may have imposed recall and social desirability biases [37]. Also, although the CSES questionnaire includes clinical skills commonly involved in providing chronic and acute patient care, it contains primarily basic skills learned in the early stages of studies. Therefore, high scores found regarding confidence in their application but with a difference in experience, might not be surprising. Moreover, since weak positive correlations were found between the research variables, it may be advisable to examine similar correlations in larger samples [38]. In addition, the current study involved students studying at six nursing schools affiliated with one major university and therefore, they might have similar characteristics. Finally, the distribution of the questionnaires was halted with the outbreak of COVID-19. The pandemic has presented healthcare systems with a challenge that could affect students’ preferences and choices of specialization.

## Conclusions

This study provides insights into personality traits and specialization choices of nursing students in the final stages of their studies and taking their first steps in the labor market. The results add to the important knowledge of managing nursing personnel, as nursing systems should measure personality traits in order to match this variable with different nursing areas. This study helps illuminate factors that attract the younger generation to the various areas of specialization. The fields of geriatrics and mental health are still failing to attract students to choose them as preferred fields of specialization. The current findings may serve as a basis for the development of a tool for occupational guidance, designed to rank students’ suitability for each area of specialization and thus help them choose what best suits their character. Matching personality traits to the field of specialization can reduce subsequent job dissatisfaction with one’s chosen field and with the nursing profession in general. In addition, a career development path, i.e., clinical expertise, must be developed and applied. Health systems that face the challenge of growing chronic morbidity due to the aging population are assimilating new roles in the health system, such as that of clinical specialist nurse.

In Israel, 46% of the RNs have completed an advanced training course for specializing in one of the 26 areas [3]. In recent years the Ministry of Health has been promoting “Nurse specialist” training in eight fields that include the following: diabetes, rehabilitation, geriatrics, supportive care, premature infants, surgery, community health, and policy -administration. At present, only less than 1% of RNs have completed this “Nurse specialist” training [3]. Regarding geriatrics, although there is a clinical expertise in this field, expert positions and regular work regulations have not yet been institutionalized. Therefore, the effect of the novel expert position on the prestige of the field and on attracting future staff is not yet evident. Moreover, at present, an expertise program in the field of mental health has not yet been established in Israel. On the policymaking level, we recommend that different measures be considered to help raise the prestige of geriatrics and mental health, such as providing incentives to nurses working in these fields.

### Electronic supplementary material

Below is the link to the electronic supplementary material.

Supplementary Material 1

## Data Availability

All data generated or analyzed during this study are included in this published article. The datasets during and/or analyzed during the current study are available from the corresponding author on reasonable request.

## Abbreviations

RN: Registered Nurses
BFI: Big Five Index
APRNs: Advanced Practice Registered Nurses
CSES: Clinical Self-Efficacy Scale
SD: Standard deviation
